# Treatment of Vertical Maxillary Excess and Skeletal Class II Malocclusion: A Case Report

**DOI:** 10.7759/cureus.65367

**Published:** 2024-07-25

**Authors:** Komal J Agrawal, Khyati Gupta, Prachi Khandelwal, Vaibhav Pipare, Anushka Jain, Khushee Raisoni

**Affiliations:** 1 Orthodontics and Dentofacial Orthopedics, Sharad Pawar Dental College and Hospital, Datta Meghe Institue of Higher Education and Research, Wardha, IND

**Keywords:** high-pull headgear, overbite, overjet, class ii malocclusion, beggs wrap

## Abstract

Class II malocclusion is a recurrent problem that may occur at a young age. If treated initially, the malocclusion can be corrected by redirecting the growth without invasive modalities and avoiding orthognathic surgeries. A female patient aged 10 years three months came to the department of orthodontics having a complaint of upper front teeth placed forwardly, diagnosed with skeletal class II due to retrusive mandible and vertical maxillary excess with hyper divergent growth pattern with increased anterior facial height, with Angle’s molar class II division 1 malocclusion, increased overjet of 13 mm and overbite of 7 mm, acute nasolabial angle, deep mentolabial sulcus, and hyperactive mentalis. It was treated using an activator with medium-high-pull headgear (modified Herren activator) passing through the maxillary center of resistance. A fixed mechanotherapy with high-pull headgear was given using the anterior inclined plane acrylic plate in the maxilla and McLaughlin, Bennett, and Trevisi (MBT). Begg’s wrap was used for the retention plan. This case report shows the significance of proper treatment results obtained due to correct identification and planning in treating malocclusion. This case report shows the significance of proper treatment results obtained due to correct identification and planning in treating malocclusion.

## Introduction

A malocclusion is a group of dental and facial abnormalities in the position of the teeth and jaws affecting oral health and facial shape and function, which requires orthodontic management [1]. A maxillary overgrowth, an underdeveloped mandible, or both can lead to class II malocclusion. Lack of mandibular development, distal position of the mandible, inherited, intrauterine molding, trauma to the mandible, and muscle dysfunction can be the etiological features of malocclusion. Although mandibular skeletal retrusion is probably a frequent typical occurrence, habits like mouth breathing can also lead to such malocclusions. Among developing individuals, skeletal class II malocclusion accompanied by mandibular deficiency and maxillary excess is a serious skeletal issue [2]. Numerous characteristics, including dentoalveolar growth, maxillary and mandibular growth patterns, and facial anatomy, are linked to the nature of a class II malocclusion [3]. Different aspects of these characteristics must be taken into account when treating malocclusions. The clinical and radiographic findings of a 10-year-old female patient, with forwardly placed upper front teeth, clearly showing the presence of a skeletal class II malocclusion are presented in this case report. A clinical examination revealed that there is a maxillary protrusion vertically and a retrusive mandible. Furthermore, the patient had Angle’s class II division 1 malocclusion with an overjet of 13 mm and overbite of 7mm. When managing adult individuals with skeletal-based problems, a combination orthognathic-orthodontic procedure is a particularly successful way. For many of these patients, surgery could prove to be an option because of economic as well as health issues [4]. Class II division 1 malocclusion improvement with high-pull headgear activator combination therapy generally results in improved condylar, glenoid fossa remodeling and mandibular posterior teeth, restricting mesial along with vertical shifting of maxillary teeth, and improved musculature pattern [3,5,6]. An activator with headgear appliances employed together is one of the very popular functional appliances for treating sagittal advancement of the mandible under vertical control. This device causes elevator and protractor muscles to contract more while simultaneously causing the retractors to relax and extend. As muscles adjust to new functional pressures, this results in a more advantageous muscle pattern and a shift in bone structures [2]. Treating such complicated anteroposterior and transverse dental patterns should not be the orthodontic solution alone. However, it should involve a multidisciplinary team of orthodontists, periodontists, and/or surrogate surgeons to get the best functional and aesthetic results [7]. This case report intends to explain in detail the diagnostic process of a case like this, the factors involved in treatment planning, and the orthodontic procedures used to manage such complex dental and surgical anomalies.

## Case presentation

**Figure 1:**
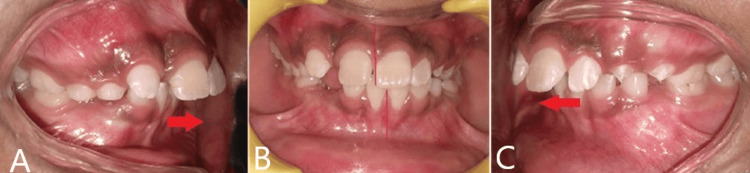
Images showing (A) overjet, (B) midline shift, and (C) overbite

A 10-year, three-month-old female prepubertal patient visited our department complaining of forwardly placed upper front teeth. On examination, she was diagnosed with skeletal class II due to vertical maxillary excess and retrusive mandible. It was noticed that there is a hyperdivergent growth pattern with increased anterior facial height. The molar relation was found to be Angle’s molar class II division 1 malocclusion bilaterally. There was an increase in overjet by 13 mm and overbite by 7 mm (Figure 1).

**Figure 2:**
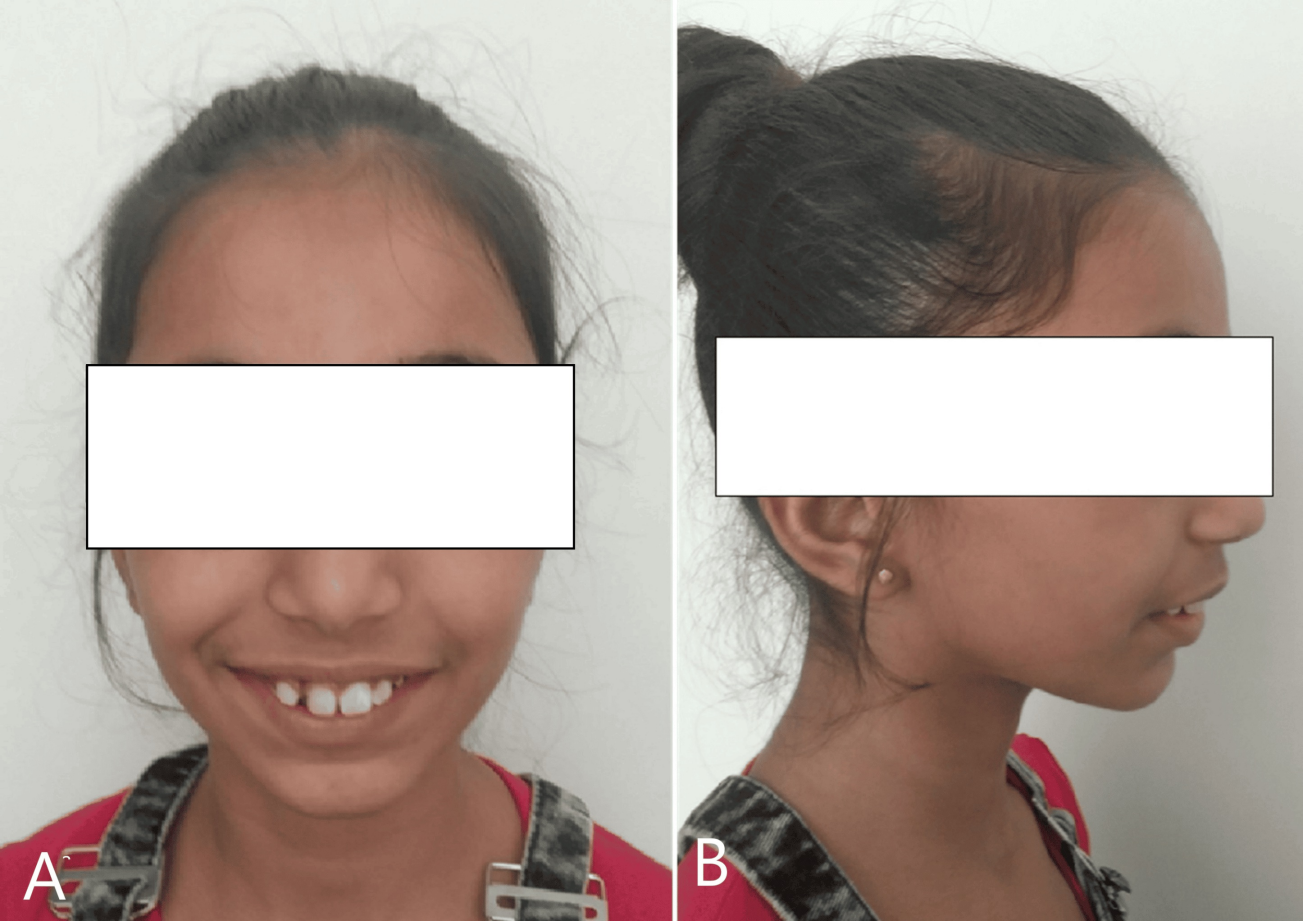
Extraoral pretreatment photograph (A) Front profile (B) Lateral profile

An acute nasolabial angle, deep mentolabial sulcus, and hyperactive mentalis muscle were detected. On general examination, it was seen that lips were incompetent with a gummy smile and retruded chin. On clinical examination, extraoral features like the head profile were dolichocephalic, the facial form was laparoscopic, and a convex facial profile was seen (Figure 2).

**Figure 3:**
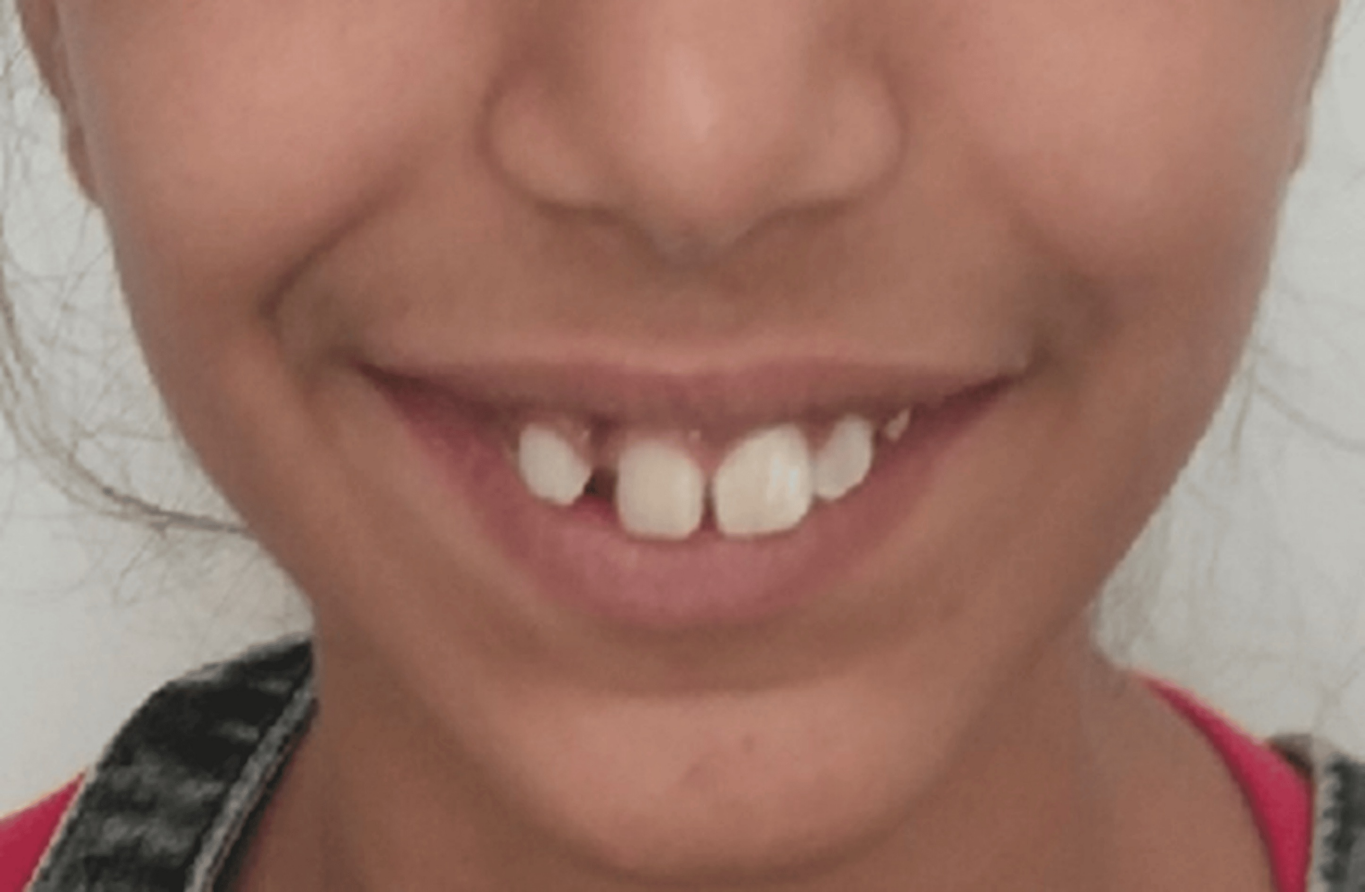
Smile analysis

Other diagnostic findings were deep mentolabial sulcus and acute nasolabial angle. On smile analysis, it was found that there is a high lip line, non-consonant smile arc, upper midline coinciding with lower midline, flat upper lip curvature, and incisor visibility during smile is 10 mm + 3 mm of gingival visibility and rest is 8 mm. With these findings, grade 1 fluorosis is present (Figure 3).

**Figure 4:**
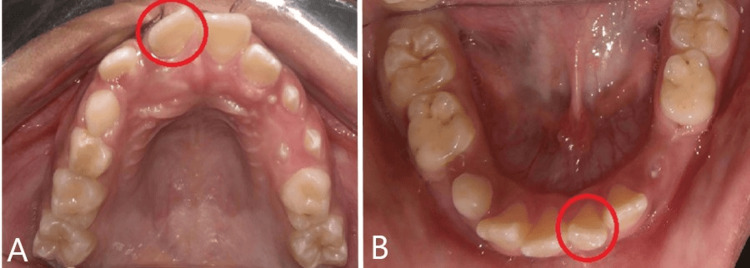
The marked area shows the mesially rotated and distally rotated lateral (A) Intraoral upper arch (B) Intraoral lower arch

In the upper arch, it was seen that there is a U-shaped symmetrical arch, mesiobuccal rotation of the right central incisor, and the presence of deciduous teeth. According to Nance analysis, the sum of lower incisors is 28.5 mm, and the Leeway space is 1.8 mm. In the lower arch, it was seen that there is a mild crowding of 2.5 mm, a curve of speed of 2 mm, a Leeway space of 3.4 mm, and a distobuccal rotation of lower incisors with the presence of deciduous teeth, both E and right C (Figure 4).

**Figure 5:**
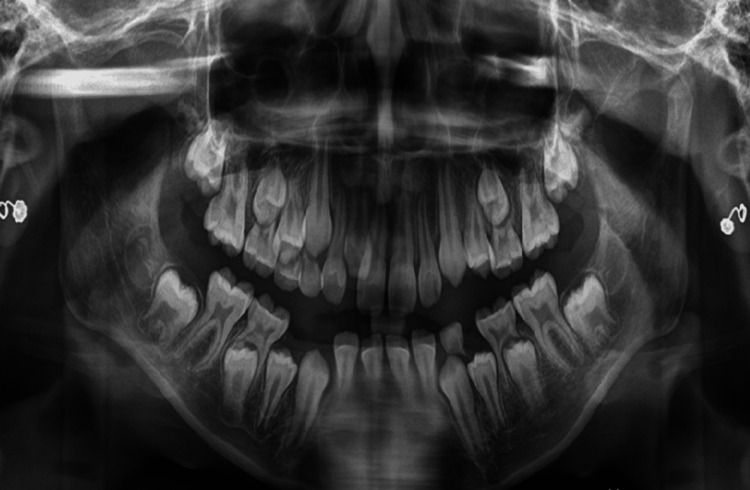
Pre-treatment orthopantomogram

According to functional analysis, the centric relation-centric occlusion discrepancy is 3 mm, the freeway space is 3 mm, and lateral occlusion is present. The radiographic analysis through an orthopantomogram shows a palatal blocked right lateral incisor. All the mentioned findings can be appreciated here (Figure 5).

Cephalometric analysis

**Figure 6:**
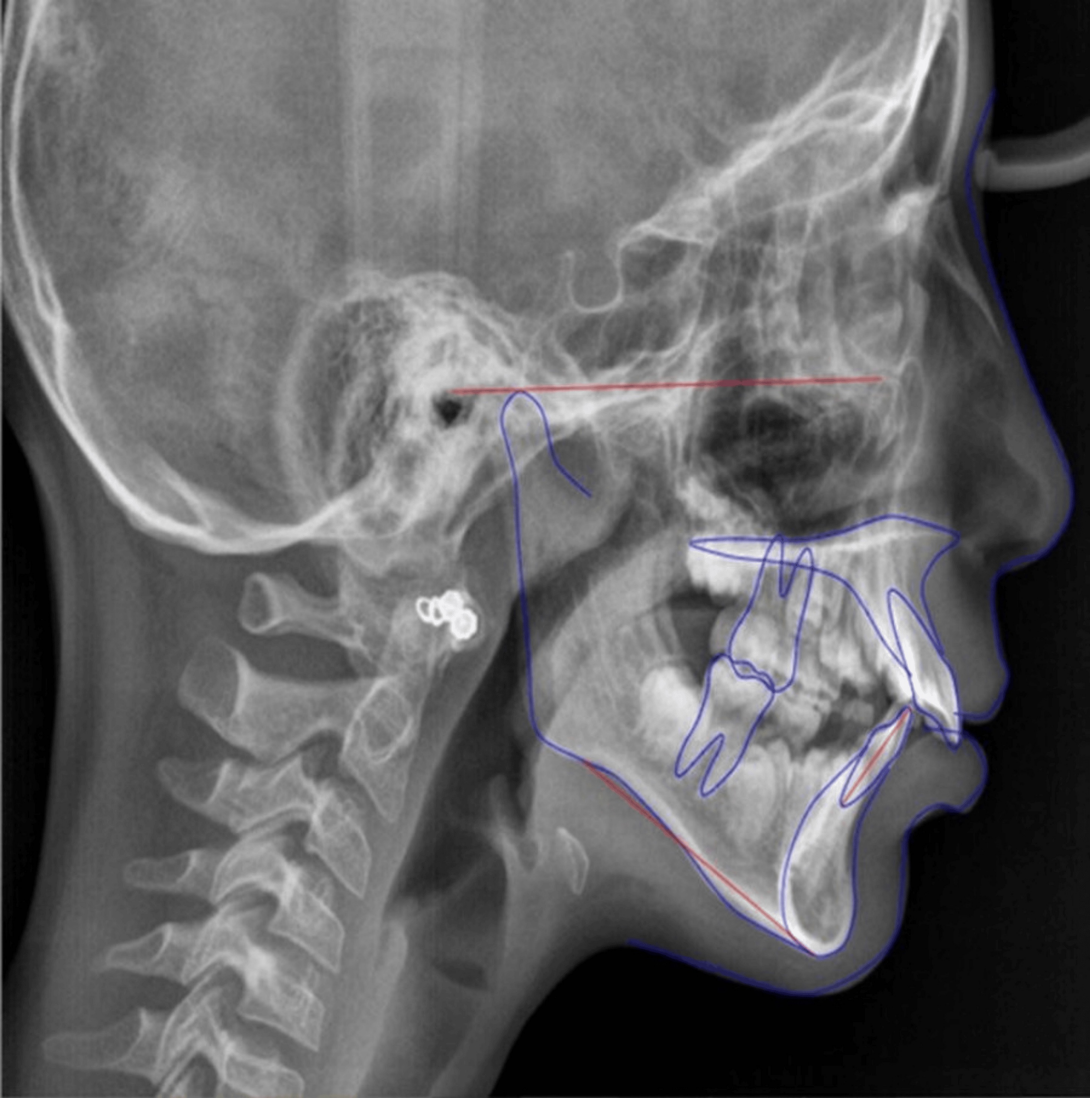
Pre-treatment cephalometric analysis

In the pretreatment analysis of the patient, it was found that there is a vertical growth pattern, orthognathic maxilla, retrognathic mandible, and forwardly placed upper and lower incisor with protruded upper and lower lip having skeletal class II malocclusion. The cephalometric analysis was done through the lateral cephalogram (Figure 6), and the pretreatment cephalometric findings (Tables 1, 2) were obtained.

**Table 1:** Pre-treatment skeletal parameters SNA: The angle between the sella/nasion plane and the nasion/A plane SNB: The angle between the sella/nasion plane and the nasion/B plane ANB: The angle between point A of the maxilla and point B of the mandible GO-GN: The angle between the gonion/gnathion plane and the sella/nasion plane

Skeletal parameters	Normal	Value
SNA	82°	83
SNB	80°	76°
Nasion perpendicular to point A	0 ± 2°	
Nasion perpendicular to pogonion	0-1°	
Beta angle	27-35°	20°
Effective maxillary length	74.8 mm	73 mm
Effective mandibular length	18.86 mm	85 mm
GO-GN to SN	32°	38°
Frankfort-mandibular plane angle	25°	30°
Jarabak ratio	62-65	58
Bjork sum	396	410
Saddle angle	123 ± 5°	130°
Gonion angle	128°	140°
Y-axis	66 mm	73 mm
ANB	2°	7°

**Table 2:** Pre-treatment dental parameters U1-NA: Upper incisor to nasion-A measures L1-NB: Lower incisor to nasion-B measures U1-L1: The angle between the long axis of upper incisors and the long axis of lower incisors U1-SN: The angle between the long axis of upper incisors and the sella/nasion plane L1-NB: The angle between the long axis of lower incisors and nasion-B L1-A pog: Lower incisor to pogonion-A measures

Dental parameter	Normal	Value
U1-NA	4/22	8/32
L1-NB	4/25	7/26
Incisor mandibular plane angle	90°	95°
U1-L1	130°	108°
U1-SN	102°	118°
L1-NB	25°	27°
L1-A pog	1-2 mm	4 mm
Steiner line to the upper lip	-2 mm	3 mm
Steiner line to lower lip	0 mm	5 mm
Nasolabial angle	90-100°	95°

Treatment objectives

The treatment goals are to correct the jaw relation, improve the gummy smile, get normal overjet and overbite, correct the smile and soft tissue profile, properly align the teeth, get molar and canine class I relation, correct the facial midline, and correct the competency of lips.

Treatment plan

**Figure 7:**
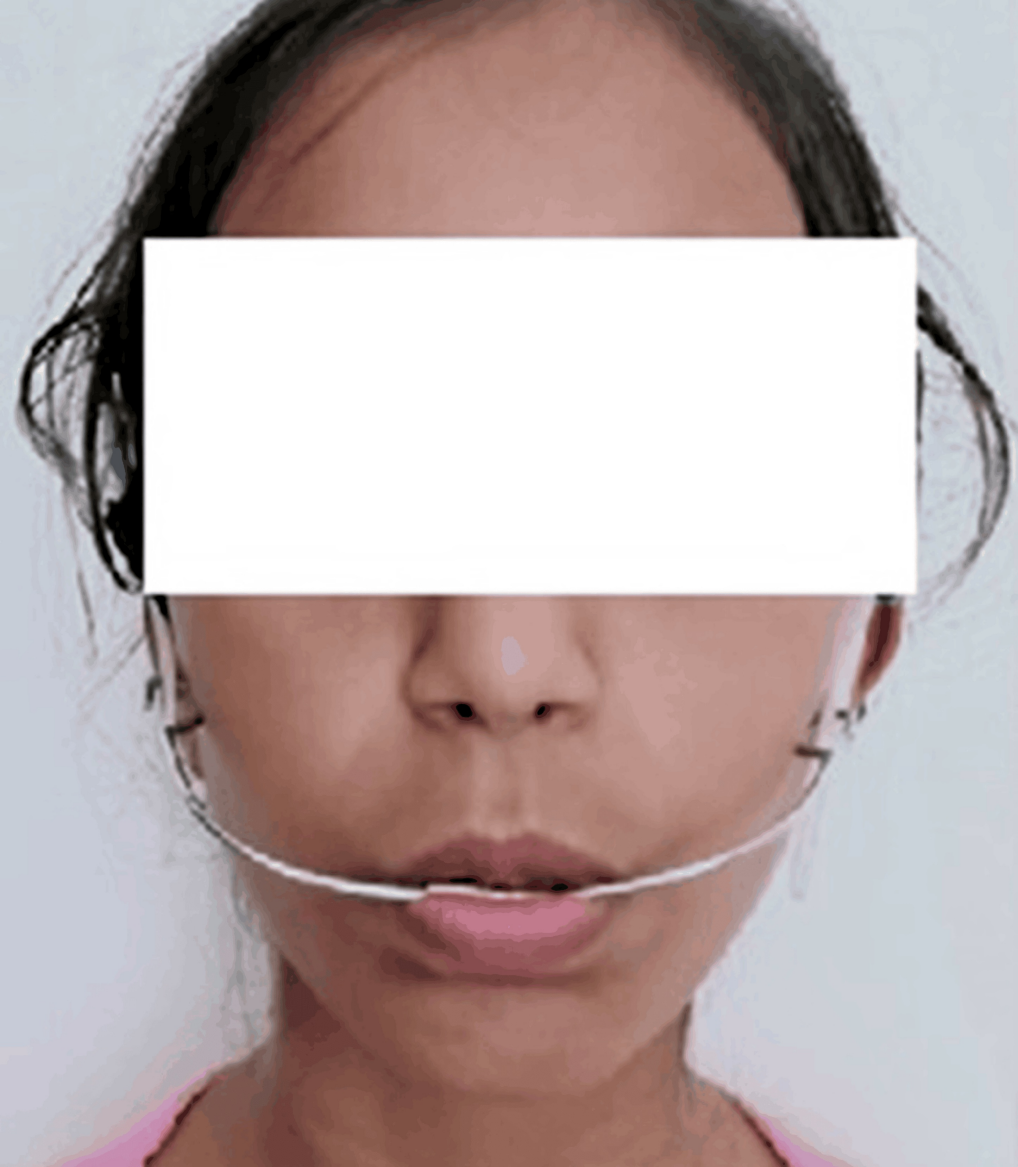
Intraoral headgear

Diagnostic tools such as cephalometric analysis and radiographic imaging were crucial in confirming the disturbance in occlusion. On completion of diagnosis, planning of the orthodontic treatment was done. The treatment plan primarily aimed to correct the chief complaint of forwardly placed upper front teeth. Once the oral prophylaxis was done, the treatment began with giving an activator with a medium-sized pull headgear (modified Herren activator) passing through the maxillary center of resistance (Figure 7). The force applied was 300 gm on each quadrant to wear 12-14 hours per day and activator 16-18 hours per day. Every month, the force was increased by 50 gm on each side, starting with 350 and up to 500 gm.

**Figure 8:**
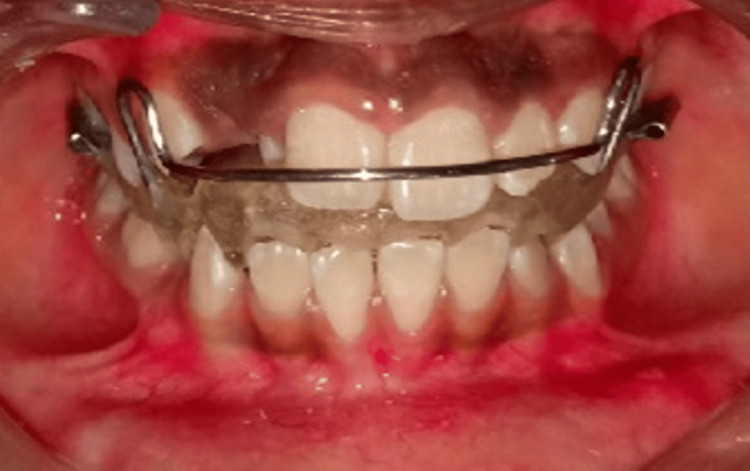
Fixed mechanotherapy

After the activator was fixed, mechanotherapy was given. An anterior inclined plane acrylic plate in the maxilla was given along with McLaughlin, Bennett, and Trevisi (MBT) of 0.022 × 0.028″ slot bracket (Figure 8).

**Figure 9:**
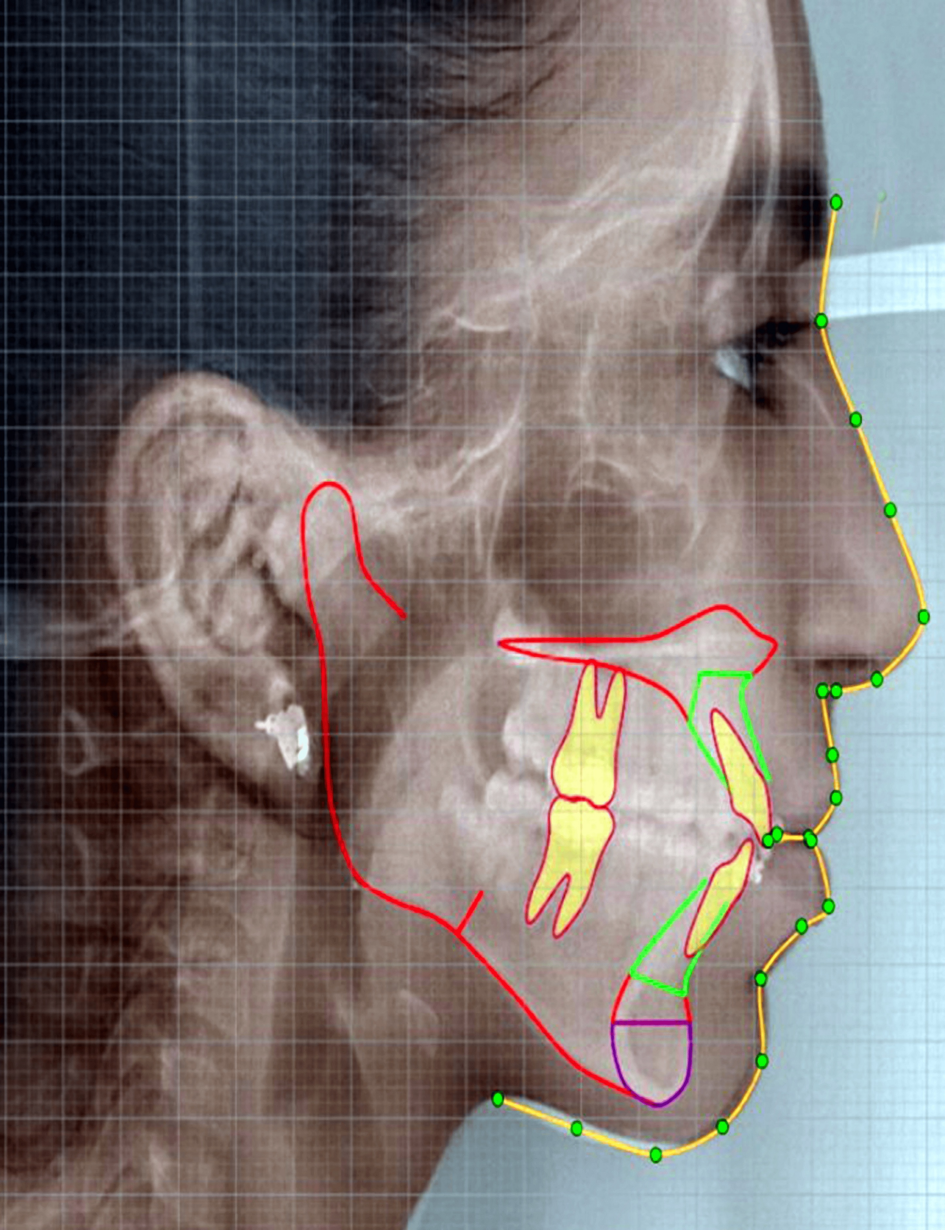
Post-treatment lateral cephalogram

**Figure 10:**
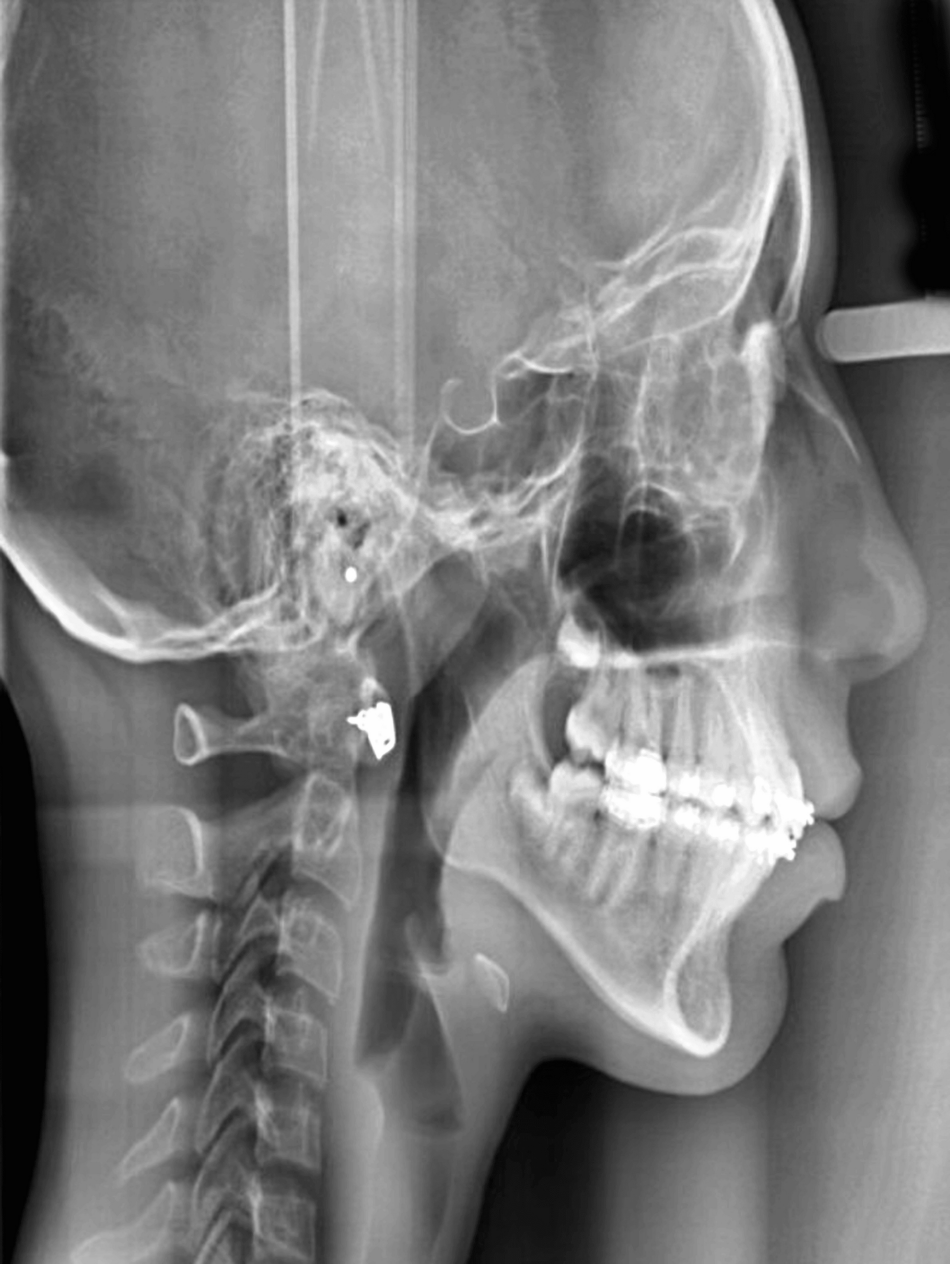
Post-treatment orthopantomogram

**Figure 11:**
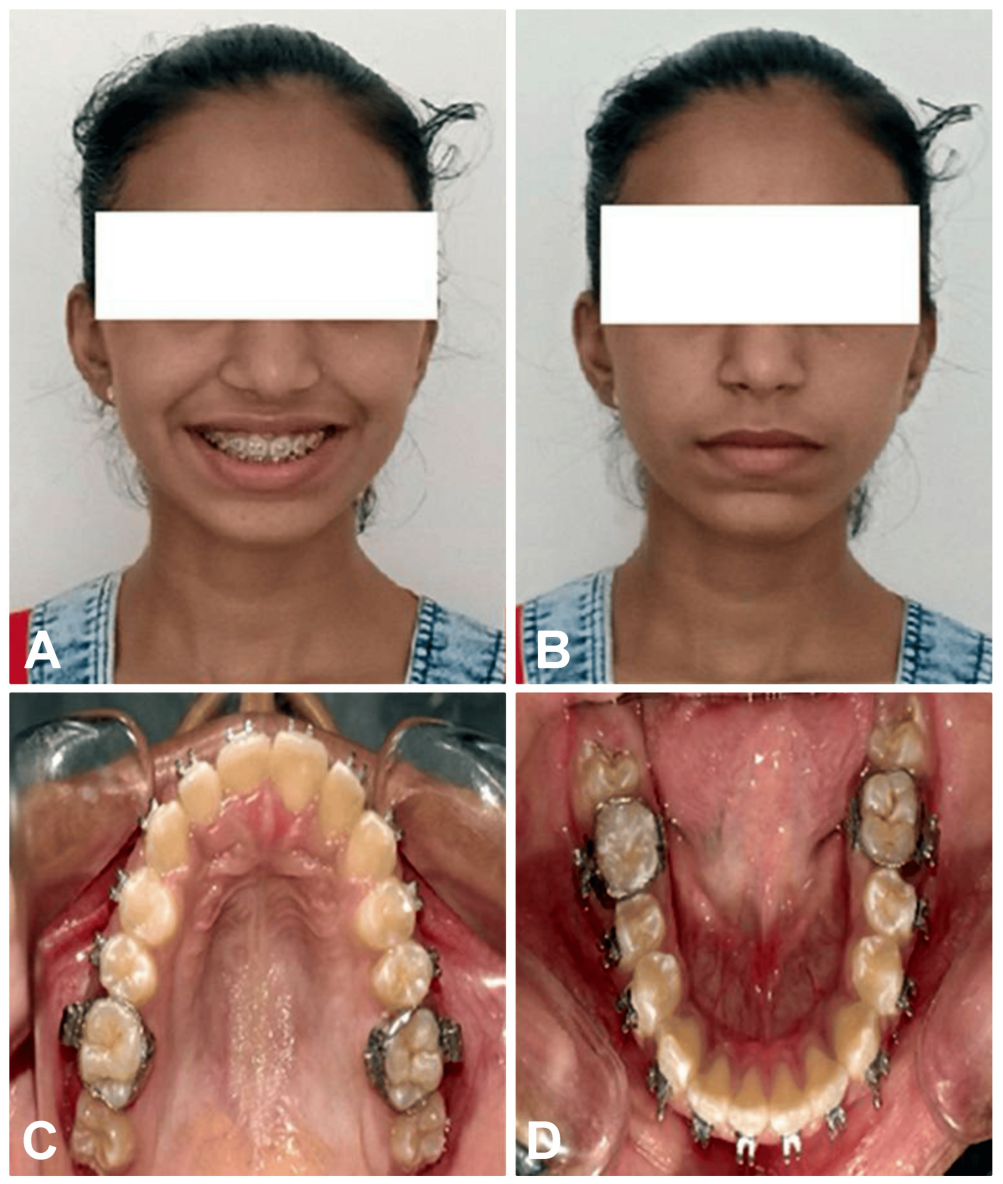
Post-treatment photos (A,B) Extraoral photo (C) Intraoral maxillary arch (D) Intraoral mandibular arch

After that, a high-pull headgear was given for nighttime wear. Lastly, a retention plan was given, incorporating a Begg’s wrap-around with an inclined plane after removing fixed appliances. The prognosis of the treatment was found to be good further. The post-treatment lateral cephalogram (Figure 9) and orthopantomogram (Figure 10) can be appreciated. The pre-treatment and post-treatment cephalometric comparison can be evaluated (Table 3). The post-treatment findings after correction were skeletal class I malocclusion, anti-clockwise rotation of the palatal and mandibular plane, increased posterior facial height, and correctly proclined and forwardly placed upper and lower incisors (Figure 11).

**Table 3:** Post-treatment cephalometric comparison SNA: The angle between the sella/nasion plane and the nasion/A plane SNB: The angle between the sella/nasion plane and the nasion/B plane ANB: The angle between point A of the maxilla and point B of the mandible FMA: Frankfort-mandibular plane angle GOGN-SN: The angle between the gonion/gnathion plane and the sella/nasion plane U1-NA: Upper incisor to nasion-A measures L1-NB: Lower incisor to nasion-B measures U1-L1: The angle between the long axis of upper incisors and the long axis of lower incisors

Parameter	Norm	Pre	Mid value	Current value
SNA	82	83	82	83
SNB	80	76	79	80
ANB	2	7	3	3
FMA	22-28	30	27	26
GoGn-SN	32	38	31	32
U1-NA	4/22	8/34	5/25	3/25
L1-NB	4/25	8/26	7/30	5/28
IMPA	90	95	100	98
U1-L1	130	108	112	128
UPPER LIP -s PLANE	-2	3	1.5	1
Lower lip -E plane	0 mm	5	3	2
Nasolabial angle	102	95	100	100
Y-axis	66	69	65	64
Jarabak’s ratio	62-65	58	63	64

## Discussion

The primary purpose of the presented case report is to present and explain the orthodontic and skeletal considerations of the condition, specifically the diagnostic findings and the guidelines in the treatment planning. Since the problem described is severe, an individualized treatment plan is required, which will involve different specialists. A 10-year three-month-old female patient came to the department with the complaint that her upper front teeth were malaligned, and on diagnosis, it was given the clinical diagnosis of skeletal class II malocclusion. An orthognathic surgery is likely required, and the prognosis of the surgery may yield the best results. They also involve the establishment of a class I relationship, reduction in the overjet and overbite, as well as functional occlusion and facial aesthetics. When performing the orthodontic treatment, fixed appliances will be applied to produce the changes necessary in the arch form and tooth position. The proposed treatment encapsulates the reduction and biomechanical alignment of the protruded upper central incisors in conjunction with the correct positioning of lower incisors to a more suitable occlusal position [1,7]. Dental documentation may also be employed to consider growth modification techniques that would provide improved treatment planning for the patient. If the skeletal malocclusion is severe, orthognathic surgery, including maxillary impaction or advancement and, occasionally, mandibular advancement, could be carried out to provide a long-lasting solution [1,8]. These surgical operations intend to establish a better balance concerning the two jaws, both practically and in terms of facial aesthetics. Correcting the acute nasolabial angle and deep mentolabial sulcus would positively affect the patient’s facial profile and smile aesthetics with orthodontic and surgical help [1,7]. The functional requirements of treatment are oriented to ensure proper occlusion of the opposing teeth, minimization of the probability of the occurrence of temporomandibular joint disorders, and the provision of the patient with lasting oral health. Various possible treatment options may be possible. Once the patient’s development is finished, the first option is orthosurgical management. The drawback of that approach was that it required a few years of waiting for the patient to recover. Moreover, the treatment plan included tooth extraction, fast palatal expanders, and high-pull headgear [4]. The second option for class II division 1 malocclusion involved extracting the maxillary first premolars using camouflage. Nevertheless, the patient’s frontal and aspect features are unlikely to be enhanced by this method of treatment. Additionally, it wouldn’t stop the maxilla’s vertical expansion [2]. As a result, this therapeutic option was enacted. As a following course of therapy, activator-headgear combination therapy was chosen to control the vertical growth of the maxillary arch and the rotation of the jaw in the backward direction, thereby improving the patient’s profile. One frequent approach to therapy for developing patients with class II malocclusion is the use of high-pull headgear in conjunction with a fixed appliance. Correcting oral malocclusion, restoring anteroposterior skeletal relationships, and preventing the deterioration of vertical skeletal relationships are the orthopedic objectives of headgear therapy [4]. An alternate twin block procedure with a higher vertical height was taken into consideration. However, the activator helmet combination was chosen because of the well-established skeletal outcomes of the combination [2]. The case report highlights several critical aspects. It challenges what to do with a growing child with vertical maxillary excess and retrusive mandible and highlights the need to time and coordinate with orthodontic and surgical specialists in the process [9]. A multidisciplinary approach can be seen. There is no doubt that the organization of treatment in the field of orthodontics, including cooperation with orthodontists and other specialists like oral surgeons, is crucial for achieving total treatment goals [10,11]. A long-term prognosis was seen. Some of the items may be the endurance of treatment results achieved and the satisfaction of the patient concerning the functional and aesthetic aspects [11].

## Conclusions

In summary, it can be said that the correction of the forwardly placed upper anterior positioning indeed leads to the attainment of the dental and skeletal changes and hence provides confidence to the management of such malocclusions through fixed mechanotherapy with the headgear. The management plan that entails using an activator headgear treatment for the changes of a severe skeletal class II with vertical maxillary imbalance is presented in this case. Fixed appliance mechanotherapy, on the other hand, assisted in the eradication of dental disparity once the desired facial transformation had been achieved. Therefore, the mode of treatment in this particular case accorded with orthodontic protocols, with significant positive changes observed in the patient’s occlusion and facial aesthetics. In consequence, it included the re-establishment of the patient’s stoma dental occlusion and the correct aesthetic facial changes. Therefore, favorable outcomes depend on continuity of awareness and adherence to the follow-up regimes. Thus, the patient could get a rather nice profile and enhance the aesthetic results by wearing activator headgear therapy. However, the best results are gotten in the long run with good follow-ups and adherence to the maintenance sequences to keep getting good results.
